# Supplementary figures and images for: Lettuce (Lactuca sativa) productivity influenced by microbial inocula under nitrogen-limited conditions in aquaponics
Source: PLoS One. 2021 Feb 23;16(2):e0247534. doi: 10.1371/journal.pone.0247534 (PMC7901782; doi:10.1371/journal.pone.0247534)

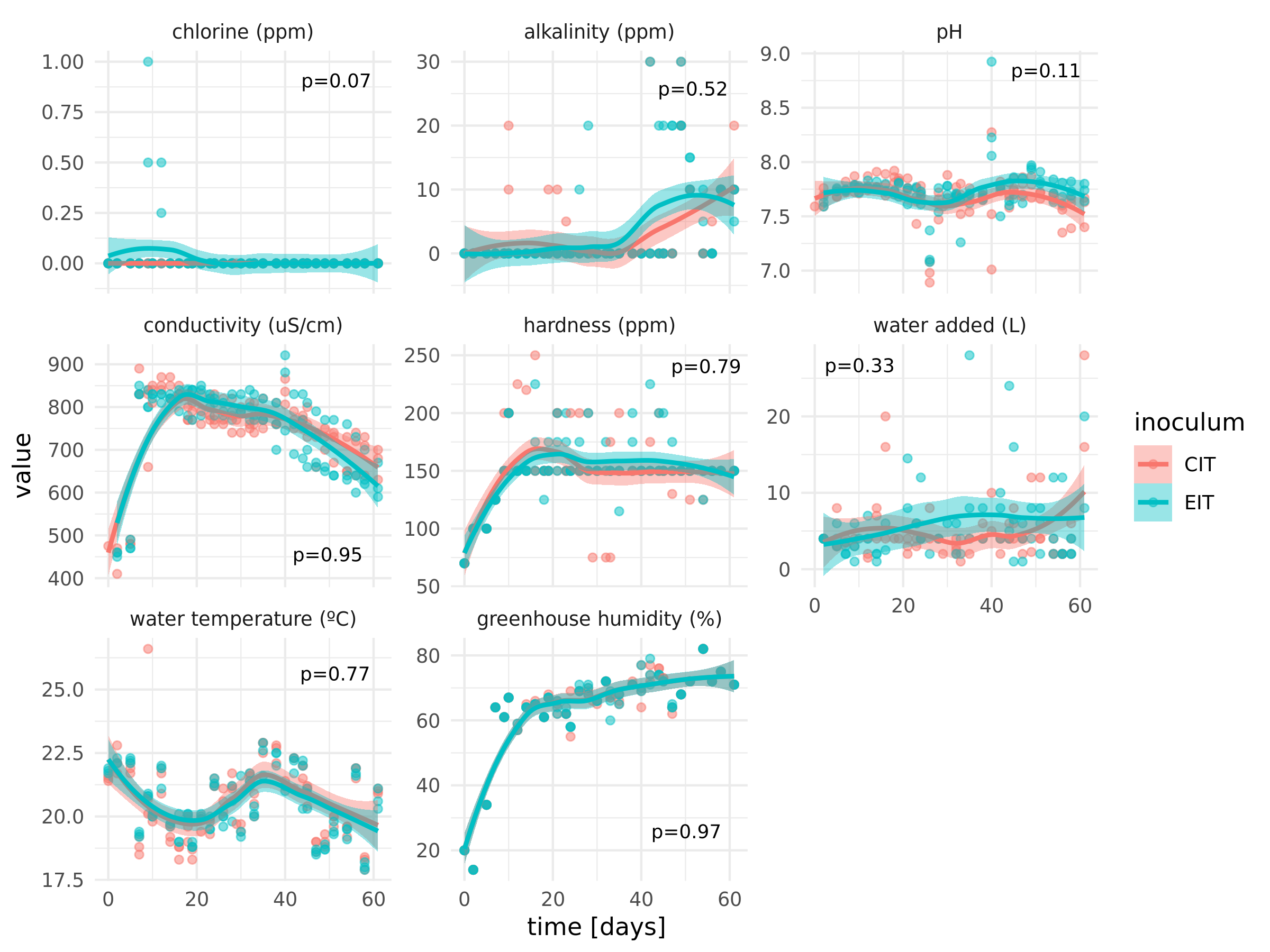

Supplement: S1 Fig — Lines denote LOESS smoothed curves for each inoculum and bands denote 95% confidence intervals of the regression. Indicated p-values were obtained from individual t-tests of CIT vs EIT systems. (PNG) [file pone.0247534.s001.png]

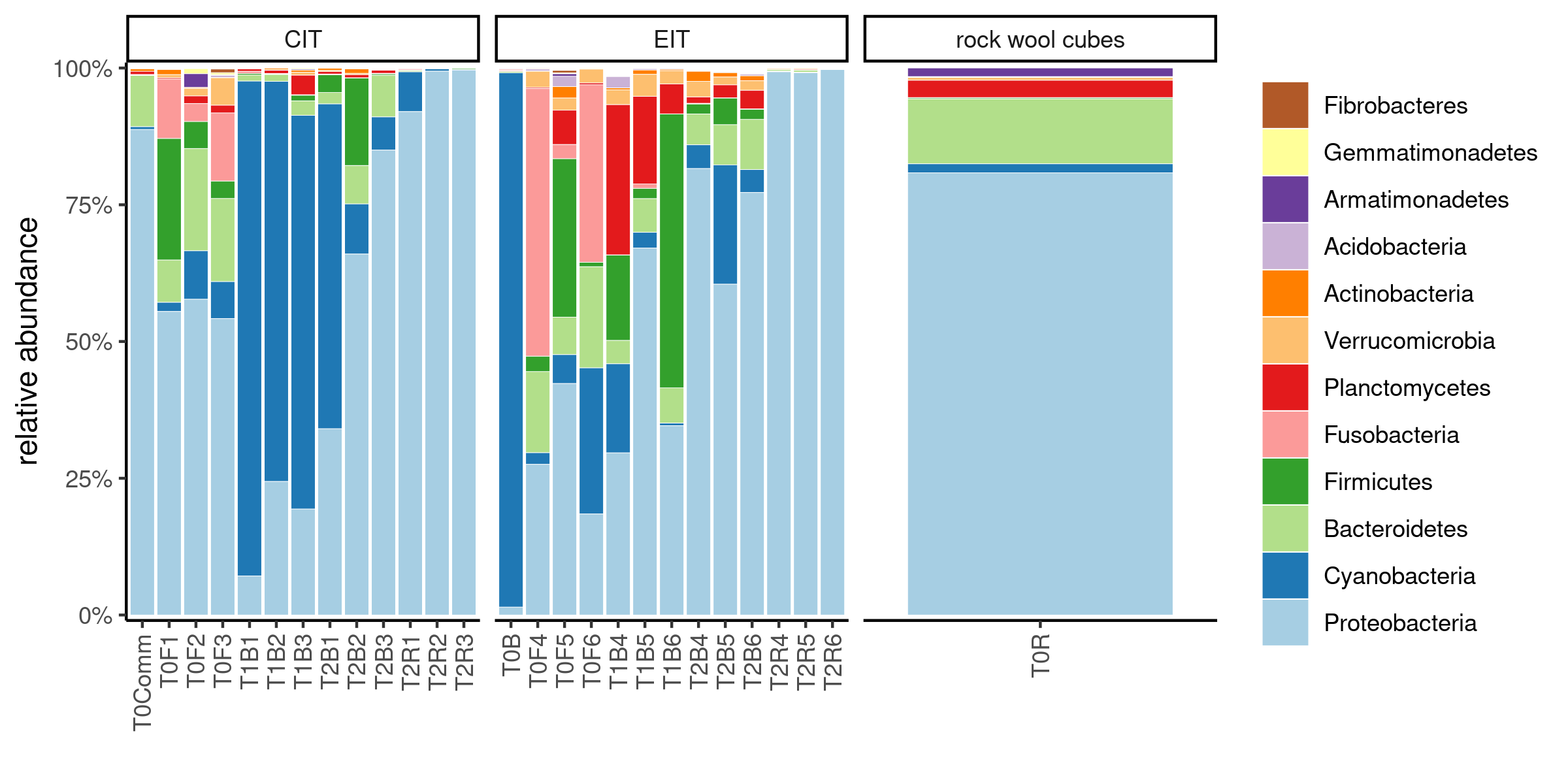

Supplement: S2 Fig — Sample names are composed of sample group ID (e.g. T1), compartment (B = biofilter, F = fish feces, R = root, Comm = commercial inoculum) and tank number (1–6). Panels denote inoculum as used in Fig 1 of the main text. (PNG) [file pone.0247534.s002.png]

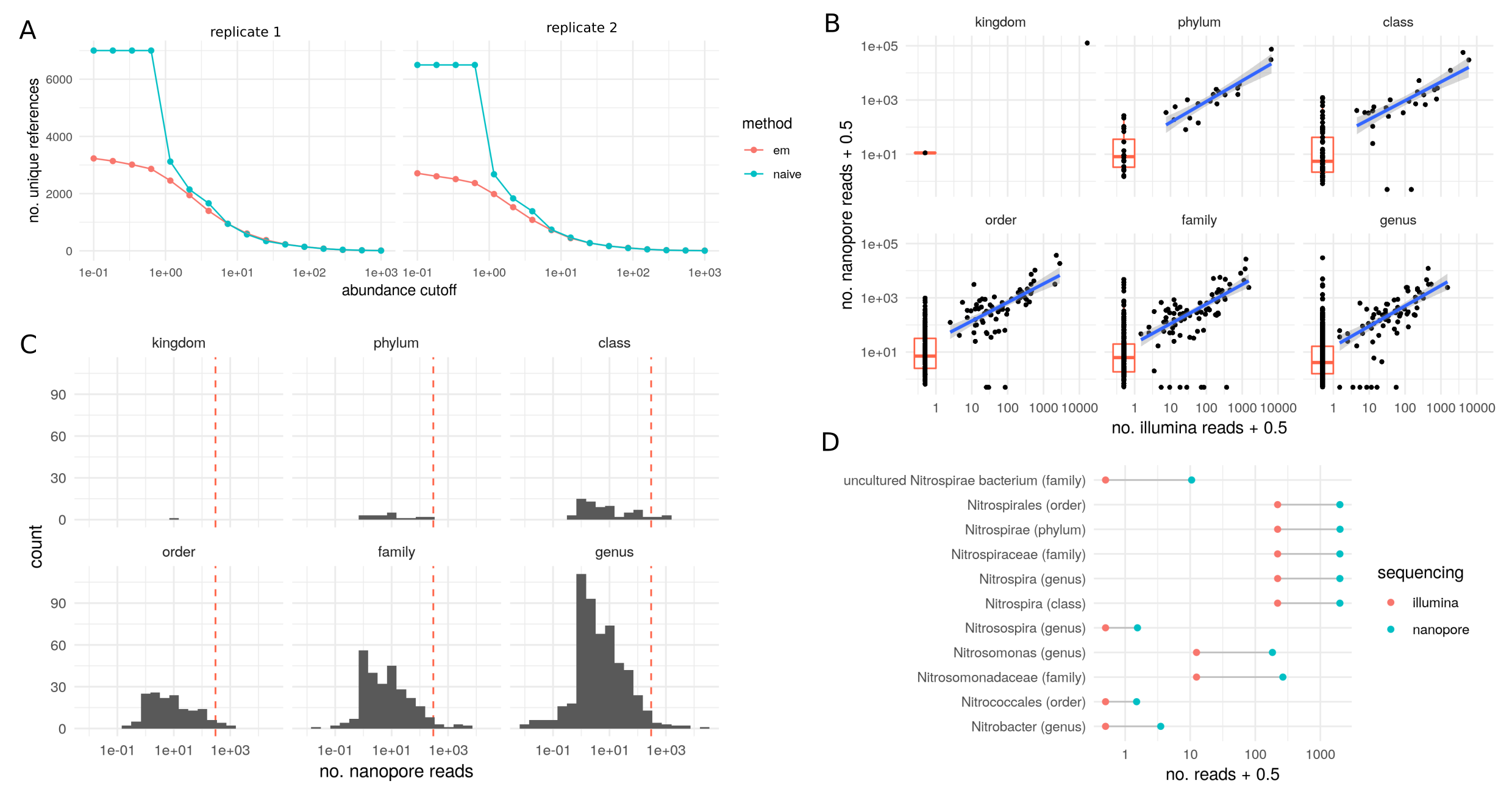

Supplement: S3 Fig — (A) In low abundances, the Expectation-Maximization (EM) algorithm identifies fewer unique references than the “naive” strategy of just selecting the highest scoring read. Each dot denotes the number of unique 16S sequences in the SILVA database that pass the abundance cutoff. (B) Abundances across sequencing protocols. Each dot denotes the abundance of a single taxon at the indicated taxonomic rank. Blue lines denote a linear regression for the taxa found in both sequencing technologies and gray errors denote the 95% confidence interval of the regression. The red boxplot summarizes the distribution of spurious mappings in the nanopore data (absent in the Illumina data). (C) Distribution of false positive mappings (mappings not observed in the Illumina data) in nanopore sequencing. The dashed red line denotes the used abundance cutoff that removed >96% of those spurious mappings. (D) Abundances of nitrifying taxa in both sequencing protocols. Dots denote the sum in the two replicates. Abundances smaller than one denote taxa not detected in Illumina sequencing. (PNG) [file pone.0247534.s003.png]

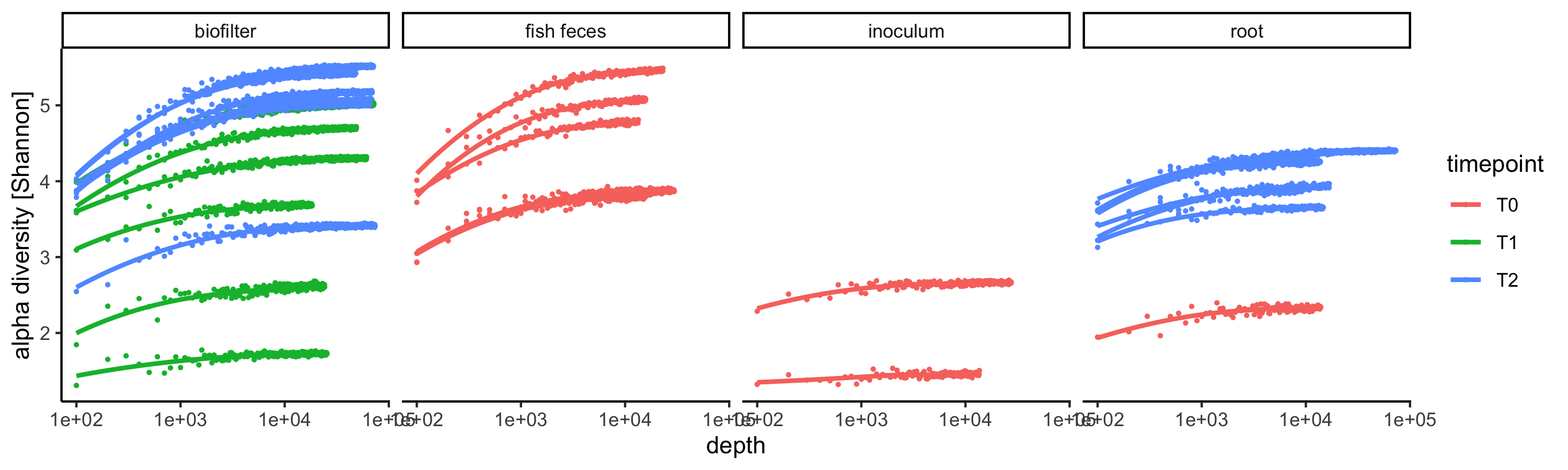

Supplement: S4 Fig — Points denote alpha diversity estimate (Shannon index) after subsampling to the specified depth. Lines denote LOESS smoothing regression lines for each individual sample. Colors and panels denote sampling time point and aquaponics system compartment, respectively. Endpoint of each curve denotes the actual depth of each sample. (PNG) [file pone.0247534.s004.png]
